# Macro Photography as an Alternative to the Stereoscopic Microscope in the Standard Test Method for Microscopical Characterisation of the Air-Void System in Hardened Concrete: Equipment and Methodology

**DOI:** 10.3390/ma11091515

**Published:** 2018-08-23

**Authors:** Fernando Suárez, José J. Conchillo, Jaime C. Gálvez, María J. Casati

**Affiliations:** 1 Departamento de Ingeniería Mecánica y Minera, Universidad de Jaén, Campus Científico-Tecnológico de Linares, Cinturón Sur s/n, 23700 Linares (Jaén), Spain; fsuarez@ujaen.es; 2 Departamento de Ingeniería Civil: Construcción, E.T.S de Ingenieros de Caminos, Canales y Puertos, Universidad Politécnica de Madrid, C / Profesor Aranguren, s/n, 28040 Madrid, Spain; jj.conchillo@upm.es; 3 Departamento de Vehículos Aeroespaciales, E.T.S. Ingeniería Aeronaútica y del Espacio, Universidad Politécnica de Madrid, Plaza Cardenal Cisneros 3, 28040 Madrid, Spain; mariajesus.casati@upm.es

**Keywords:** freezing and thawing, concrete, durability, spacing factor, pore characterization

## Abstract

The determination of the parameters that characterize the air-void system in hardened concrete elements becomes crucial for structures under freezing and thawing cycles. The ASTM C457 standard describes some procedures to accomplish this task, but they are not easy to apply, require specialised equipment, such as a stereoscopic microscope, and result in highly tedious tasks to be performed. This paper describes an alternative procedure to the modified point-count method that is described in the Standard that makes use of macro photography. This alternative procedure uses macro-photographic images that can be obtained with a quite standard photo camera and it is successfully applied to a large set of samples and presents some advantages over the traditional method, since the required equipment is less expensive and provides a more comfortable and less tedious procedure for the operator.

## 1. Introduction

The lifetime increment of structures emphasises the importance of durability in cement-based materials, thus becoming one of the most important aspects in design, construction and maintenance of civil structures. Probably, the phenomenon of freezing and thawing cycles is the most destructive environmental agent for cement-based concrete structures under continental climate conditions [1]. In open environments where freezing and thawing cycles are common, cement-based materials are frequently used in the construction of infrastructure elements, such as road pavement, covering canals, bridge decks, and sidewalks. The durability of these structures can be jeopardised, depending on the frequency of these cycles, by the expansion and contraction processes that take place during the freezing and thawing of the liquid solution, which is inside the cement-based material. This mechanism induces pressure changes, causing a fatigue process at a microstructural level of the material and eventually leading to collapse [2].

The durability and performance of cement-based materials depend mainly on the type of environment, the curing and dosage of the volume of cement, sand, aggregate, and water used in a mixture, as well as on its air content [3]. As stated by Powers in 1945 [4], the properties that are related to air voids in hardened cement-based materials have an influence on the durability and performance of structures under freezing and thawing cycles. Depending on the bubble-size distribution and the spacing of the air-void system, the durability of the cement-based materials is affected positively or negatively during these cycles [5]. 

This dependence is explained by the internal pressure variations that take place during expansion and contraction cycles that are provoked by state changes of the solution. These pressure variations may be relieved in a proper air-void system, allowing the liquid phase to flow towards the nearest air voids, while if the air voids are not well connected and according to Darcy’s law, this flow may induce pressure variations in the cement-based material due to the low permeability of the cement paste [6].

Furthermore, the characterization of the origin, size, shape, abundance, and connectivity of the voids provides information about the compressive strength, resistance to destruction by cycles of freezing and thawing and resistance to chemical attack on the reinforcing steel and the cement paste [7]. The ASTM C457 test [8] is one of the standards used to determine the parameters of the air-void system in hardened concrete materials. The earlier referenced Powers developed a model to simplify the air-void system by using the idea of the spacing factor [4,9,10], identified by $\bar{L}$, which is an index that is used to define the effectiveness of the air-void system as a contributor to frost resistance by taking into account the total air content estimated, the total air-free paste content, and the characteristic void size on the air voids spatial distribution [11]. The model is based on two ideas [6,11], which can be briefly described, as follows:

- The various size voids in the real system are replaced by single-size voids in the simplified system. The pore size in the simplified system must guarantee the same surface area and total pore volume in both systems.
- The random distribution of air voids in the real system is replaced by a geometrically regular pattern of single-size voids arranged in a uniform, three-dimensional grid in the simplified system.

Therefore, the spacing factor is defined by Pigeon et al. [6,12] as the average half-distance between two air voids that corresponds to the half of the greatest distance between any two adjacent air voids, representing the distance that water or ice would have to travel through paste to escape to the nearest air void under freezing and thawing conditions [11]. An acceptable value of the spacing factor to obtain a proper behavior of conventional concretes exposed to alternating cycles of freezing and thawing should be less than 250 nm [6]. The methodology and the relevant equations to find the spacing factor are described by the ASTM C457 [8]. The ASTM C457 procedures are based on the inspection of the surface of a polished cross-section cut from a hardened concrete slab using a microscope and the later computation of the parameters by using a set of given equations. This standard describes the two procedures that follows: Procedure A: the linear traverse method; and, Procedure B: the modified point-count method. Both methods are tedious; especially the first one, and they require specific equipment to be performed. 

In the past, several researchers have proposed alternative methods in order to reduce the time and provide a reasonable automation of the process. A comparative overview of some of them can be found in [13], concluding that they provide good results and allow rapid evaluation of freeze-thaw durability of concrete. In some cases, the alternative method consists of developing a specifically designed and built automatic system, leading to a considerable time reduction and making the process easier [14,15]. In other cases, the alternative method makes the process more comfortable for the operator and reduces the cost of the equipment by using, for example, a flatbed scanner instead of a stereoscopic microscope [16,17,18]. Other approaches are focused on using image processing software to study the air-entrained system of concrete [19]. All these methods analyse lapped concrete surfaces, prepared as described at the ASTM C457, and usually treat them by colouring them with marker pens (e.g., [14]) or ink solutions (e.g., [16]), and filling the voids with white powder (usually barium sulfate or silicon carbide) to make voids identification easier.

In this paper an alternative methodology for the modified point-count method is described, which makes use of macro-photographic images instead of a stereoscopic microscope to observe the surface of analysis. This methodology does not require additional preparation of the sample, reduces the equipment cost and makes the procedure easier to perform. It has been compared with the traditional method by analysing a large set of samples, both providing similar results; therefore, the proposed methodology used here can be considered as a reliable alternative to that described at the ASTM C457.

## 2. Proposed Methodology Based on Macro-Photographic Images

In this section, the equipment needed is presented and the proposed alternative methodology is described.

### 2.1. Equipment

**Figure 1:**
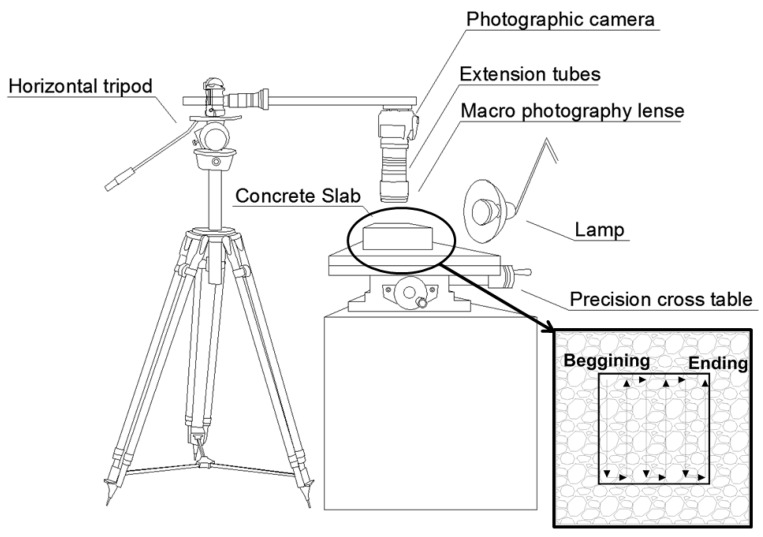
Experimental setup for the shooting of macro-photographic images of the surface analysed. In the corner of the picture, an example path for obtaining macro-photographic images covering the whole area of analysis.

The following list describes the equipment used by the methodology:

- Photographic camera with life-view shooting mode. While the life-view mode is not strictly necessary from a technical point of view, as later will be observed, it makes the procedure significantly easier to perform.
- Macro photography lenses and extension tubes these elements may vary from one piece of equipment to another. In fact, if a sufficiently high-quality macro photography lens is available, the extension tubes may be unnecessary. The extension tubes are merely an option to define a less expensive way of obtaining macro-photographic images.
- Remote-shutter camera. Although this element is also not strictly necessary from a technical point of view, it helps to guarantee a correct capture of the whole surface analysed, especially when the macro-photographic images are obtained by using extension tubes, which reduces the lighting on the specimen very much and makes it more prone to obtain blurry images.
- Horizontal tripod. Any solid enough tripod is valid, on the condition that it allows for the camera to be placed in a horizontal position, as shown in Figure 1.

- Precision cross table. It must provide a displacement range in both directions sufficient to cover the whole area of analysis.
- Spirit level or similar levelling device.
- Lamp.
- Computer-aided design (CAD) software.

### 2.2. Methodology Description

The following methodology does not provide a different approach from that described in the Standard as Procedure B, the modified point-count method; it only adapts it to make it less expensive and more convenient and practical. Therefore, the initial steps are common and the preparation of samples the same.

**Figure 2:**
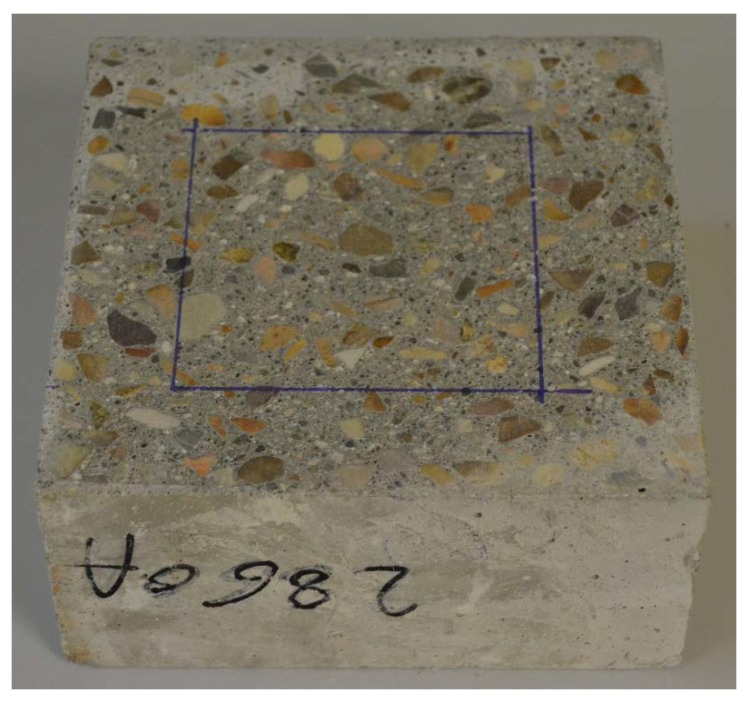
Surface of analysis drawn on the polished surfaces of a cubic sample.

**Figure 3:**
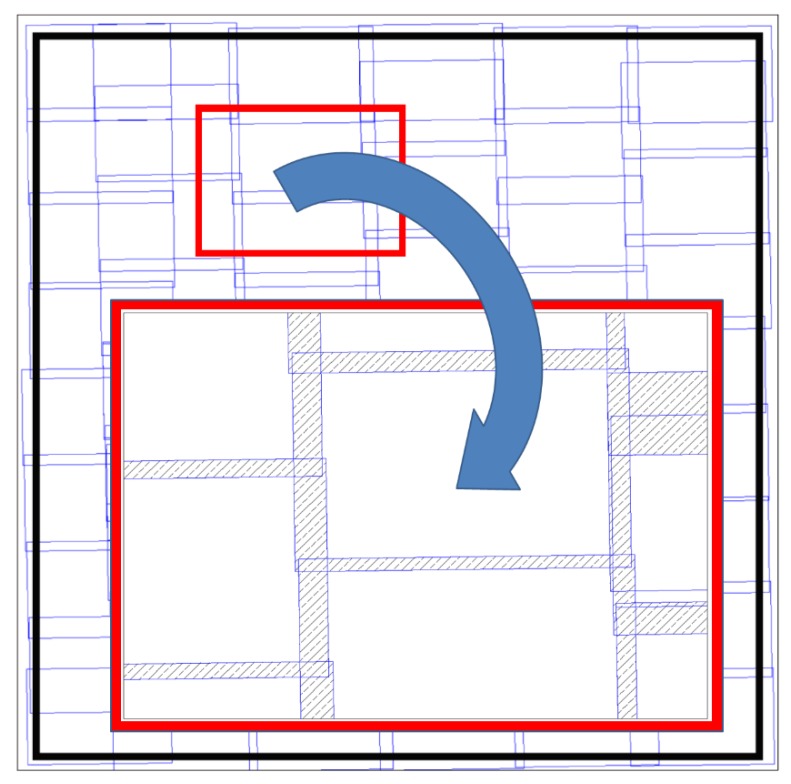
Overlapping borders of macro-photographic images covering a whole area of analysis. The black rectangle corresponds to the rectangular area of analysis marked on the polished surface.

**Figure 4:**
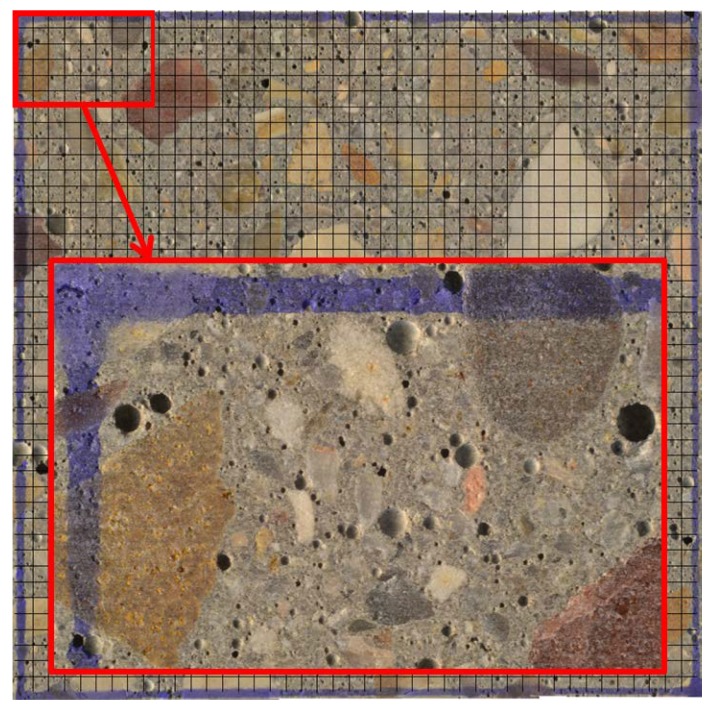
Grid superimposed on the final assembled image of 8.5 cm × 8.5 cm of the area of analysis.

Once the sample has been cut from concrete and the surface polished, as is required by the Standard, the following steps must be performed:

- Define the surface to be analysed by drawing a rectangle on the polished surface with a marker. The shape of this rectangle can be any, as long as it meets the requirements defined by the Standard (Table 1 of ASTM C457 [8]). An example of this step can be found in Figure 2.

- Place the prepared surface on the cross table. The polished surface must be in a horizontal position, since a minor error in levelling would result in unfocused images.
- Place the photographic camera, with the remote shutter connected and equipped with the macro lens and the extension tubes if necessary, on the tripod so that the camera is horizontal and at a right distance from the prepared surface to obtain a focused image. This step must be carefully performed to ensure that both the camera sensor and the prepared surface are parallel, if they are not, partially blurry images will be obtained.
- Adjust the lamp position so that the light beam illuminates evenly the area that the camera will record at each capture. The angle that is formed by the light beam and the prepared surface must be low to increase the contrast between the air voids and the polished plane, and therefore, to facilitate the identification of voids in a later step. This position must be fixed for each sample. It must be noted that, since the only element that moves during the process is the surface of the cross-table where the sample is placed, all of the images are obtained with the same illumination conditions, which is necessary for correctly identifying voids, paste, and aggregates later.
- Adjust the camera settings so that the captured image is focused and the voids can be easily identified.
- Place the sample, by using the screws of the cross table, so that the camera captures one of the corners of the rectangle that defines the surface to be analysed and take the first picture by using the remote shutter.
- Using the screws of the cross table (either north-south or east-west directions) the whole rectangle must be photographed by capturing pictures that must have overlapping areas for later assembling. An example of this procedure is shown in the bottom-right corner of Figure 1.
- Assemble the images by using computer-aided design (CAD) software. An image of the whole marked rectangle surface must be obtained by overlapping the thin common areas between each pair of consecutive pictures. Figure 3 shows an example of how the picture borders overlap and covers the whole rectangular area of analysis.

- Define and superimpose a regular grid on the image of the whole marked surface. This action depends on the minimum length of traverse and the minimum number of points (Table 3 of the Standard [8]). Figure 4 shows an example of such a grid.

- Analyse the resulting image to determine what is found at each intersection of the grid, air void, paste or aggregate, and the number of air voids that are crossed by the horizontal lines of the grid.

### 2.3. Computation of the Spacing Factor

Following the procedure described above, it is possible to obtain the same input data used in the methodology that is described in the ASTM C457 Standard [8] for Procedure B:

N=  total number of air voids.
$S_{t}=\;$ total number of stops.
$S_{a}=\;$ number of stops in air voids.
$S_{p}=\;$ number of stops in paste.
I=  the E-W translation distance between stops.

The concrete properties of are obtained as described by the Standard, while using Equations (1) to (7):

Total transverse length $\;\left(T_{t}\right)$:

(1) $$\;T_{t}=S_{t}\cdot l\;$$

Air Content (A) in %:

(2) $$\;A=\frac{S_{a}\cdot 100\;}{S_{t}}$$

Void Frequency (n):

(3) $$\;n=\frac{N}{T_{t}\;}$$

Paste Content (p) in %:

(4) $$\;p=\frac{S_{p}\cdot 100\;}{S_{t}}$$

Paste-Air ratio (p/A):

(5) $$\;\frac{p}{A}=\frac{S_{p}\;}{S_{a}}$$

Average chord length $\left(\bar{l}\right)$:

(6) $$\;\bar{l}=\frac{S_{a}\cdot I\;}{N}\;\mathrm{or}\;\bar{l}=\frac{A}{100\cdot n}$$

Specific Surface (α):

(7) $$\;\alpha =\frac{4}{\bar{l}}\;\mathrm{or}\;\alpha =\frac{400\cdot n\;}{A}$$

Finally, these values allow for obtaining the corresponding spacing factor by means of expression (8):

(8)  {L¯=p400·n if pA≤4.342L¯=3α[1.4(1+pA)13−1] if pA>4.342

## 3. Results and Discussion

In this section, the results that were obtained by using the Procedure B: the modified point-count method of the ASTM C457 Standard [8], which makes use of the alternative methodology proposed by applying macro photography techniques, are presented. First, the specimens are described and the results presented and discussed.

The camera used in this study has a resolution of 14.2 megapixels and to cover an area of interest of 72.25 cm^2^ (a square of sides of 8.5 cm) 54 photos were necessary for each slab, which resulted in a pixel size of 3.65 µm.

### 3.1. Specimens

Fifty-four cubic slabs have been studied according to the Procedure B: the modified point-count method of the ASTM C457 Standard [8]. These slabs entail 27 dosages (two slabs for each dosage). In addition, in order to study the repeatability and reliability of the method five of these dosages have been studied by using two concrete slabs per dosage and tested by two different operators. Finally, in order to compare the proposed method with the original methodology that was described in the ASTM C457, five specimens have been analysed with both methods.

As the maximum size of the aggregates used in the dosage of these slabs is 12 mm, the minimum surface to be observed should be larger than 65 cm^2^ [10 in.^2^], as defined in Table 1 of the ASTM C 457 Standard. The chosen area to be studied is a square with sides of 8.5 cm. In Table 3 of the ASTM C 457 Standard, the minimum traverse length (the total horizontal length of the area of study) and the minimum number of nodes are defined with respect to the maximum size of the aggregates, which in this case are 2032 mm [80 in.] and 1200 nodes, respectively. Given that the values chosen were 3444 mm for the traverse length and 1722 nodes, the separation between two consecutive nodes belonging to the same horizontal line of the framework is 2 mm.

### 3.2. Results and Discussion

Figure 4 shows one of the pictures that compose the marked surface of the concrete slab denominated C1A. This picture represents an area of 16.02 × 9.75 mm^2^ of the marked surface (8.5 × 8.5 cm^2^) that is tested.

#### 3.2.1. Results of 27 Dosages by One Operator

Table 1 presents the overall results of the dispersion of the air content (%), voids intersected/cm, paste content (%), and spacing factor for two slabs of the same dosage obtained by one operator. The standard deviation (1s) has the usual meaning, and thus, gives an idea of the variability of the results with respect to the average value. The range of two test results (d2s) can be seen as the expected maximum difference between any two values of the test results and it is equal to $2\sqrt{2}s$, with s being the standard deviation. Both the standard deviation and the range of two test results can be expressed as a percentage, which is made simply by dividing their values by the average value of the parameter under consideration (spacing factor, voids intersected/cm, air content, or paste content). Further information about the standard deviation and the range of two test results can be found in the ASTM C670 Standard [20].

By comparing these results with the estimate of average precision presented in Table 3 of the Standard [8], it can be observed that in the case of the spacing factor, the average standard deviation (1s) is equal to 7.5 < 8.0 and the range of two test results (d2s) is equal to 21.2 < 22.6. Therefore, both of them are smaller and thus can be considered to meet the precision.

#### 3.2.2. Results of Five Dosages by Two Operators

The main results obtained by two operators in the five dosages (two slabs per dosage) can be seen in Table 2 and the average precision data in Table 3.

**Figure 5:**
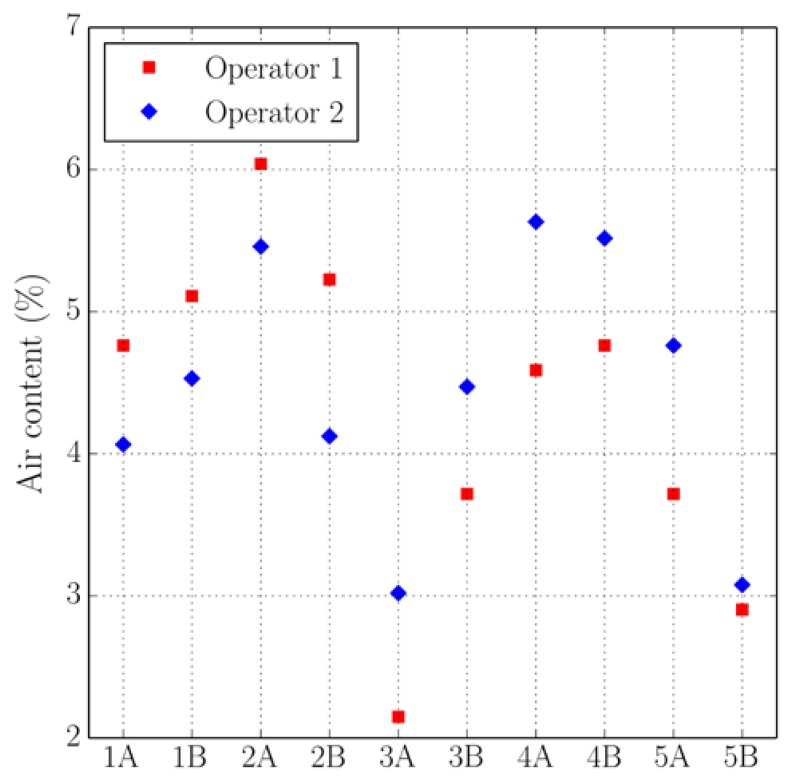
Results of the air content (%) of the slabs studied by two distinct operators.

**Figure 6:**
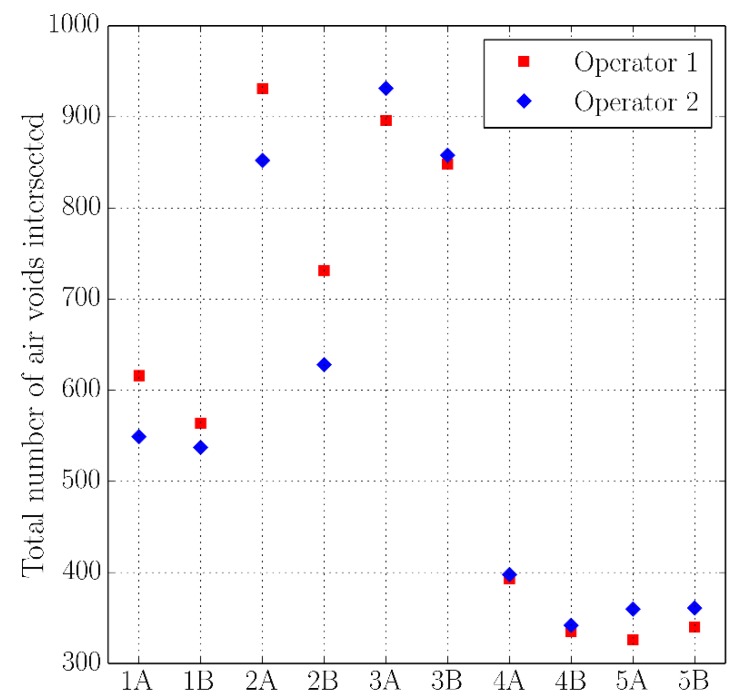
Results of the voids intersected of the slabs studied by two distinct operators.

**Figure 7:**
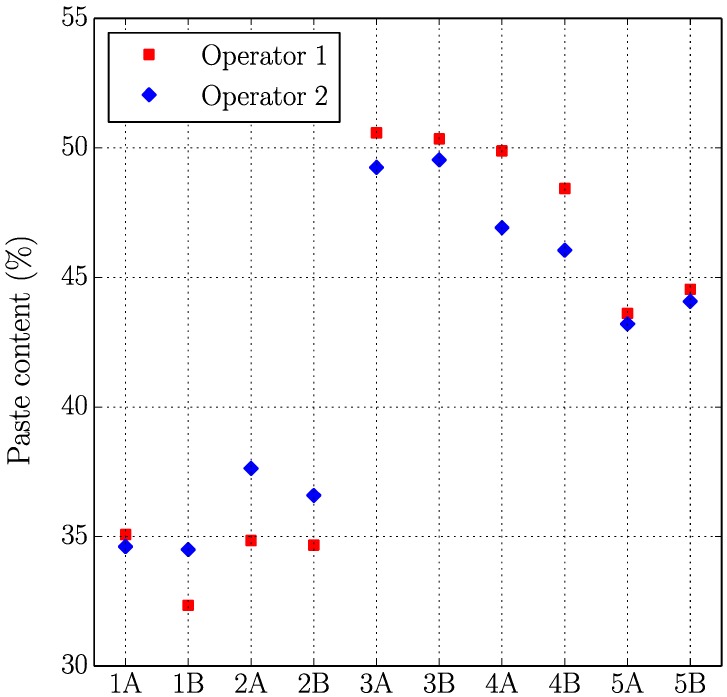
Results of the paste content (%) of the slabs studied by two distinct operators.

**Figure 8:**
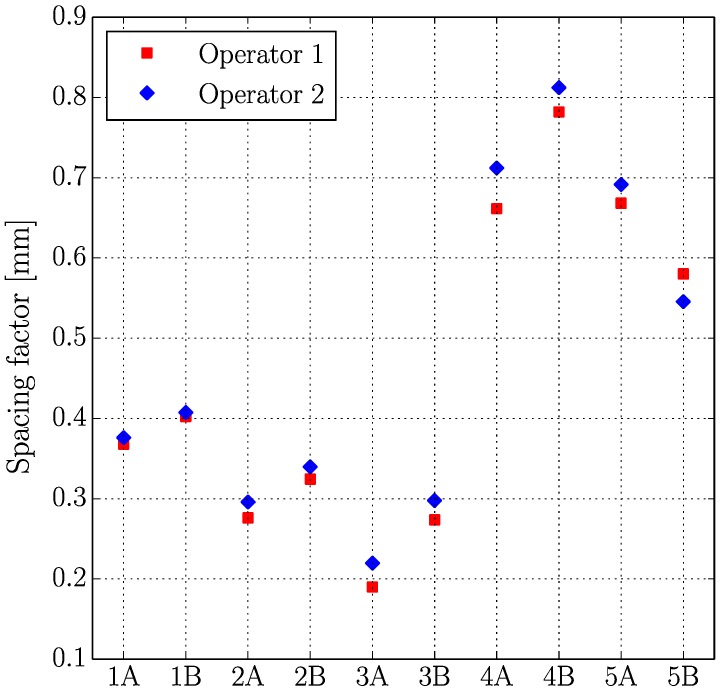
Results of the spacing factor of the slabs studied by two distinct operators.

Figure 5, Figure 6, Figure 7 and Figure 8 present the values of the spacing factor, void intersected/cm, air content (%), and paste content (%) identified in each slab by two different operators. These results show a trend related with the air voids (for air content, see Figure 5; and, for voids intersected, see Figure 6), with one of the operators tending to observe a smaller number of air voids. Possibly the reason of this is associated with the different criteria that each operator chooses when the line of the grid crosses the edge of the air void, which is somehow not totally objective. Similar differences can be observed in the case of the voids intersected/cm (Figure 6) and the number of stops in air voids. Despite this, the results corresponding to the spacing factor do not seem to be highly affected and show similar values for both operators. Bearing in mind that it is the spacing factor that determines whether a dosage of concrete is appropriate, these results show that the proposed methodology provides repetitive and reliable results. On another note, Figure 7 represents the results of the paste content (%) and shows important differences in some cases. This is because it is sometimes difficult to identify paste and aggregates when these present a similar grey colour, which, incidentally, is a problem that also arises when the procedure is performed by using the stereoscopic microscope.

Table 6 of ASTM C457 Standard [8] shows the results of a study in which the linear traverse method was applied with a magnification of 50× to two hardened concrete beam specimens with nominal air contents of approximately 3 and 6.5. These hardened concrete beam specimens were analysed by one laboratory. A pair of surfaces was sawn from the specimens for each participating laboratory; one surface was lapped in the originating laboratory, and the adjacent surface was lapped in the participating laboratory. In Table 4 and Table 5, the dispersion results of the study are compared, respectively, with those of the study [21] submitted to ASTM C457 Standard of the air content (%) and spacing factor.

Table 4 and Table 5 show that the dispersion of the results of the air content (%) and spacing factor, as assessed by distinct operators, is lower when using the method that was proposed in this paper than when using the method of the ASTM C457 Standard.

#### 3.2.3. Comparison with the Original Methodology

Five slabs were analysed by means of the original methodology that was proposed in the ASTM C457, that is to say, by using a stereoscopic microscope, and the alternative method that is proposed in this study. These slabs are of different concrete mixtures with distinct air content and air-entrainment methods; they were selected to check if the methodology agrees well with the traditional method on different cases.

**Figure 9:**
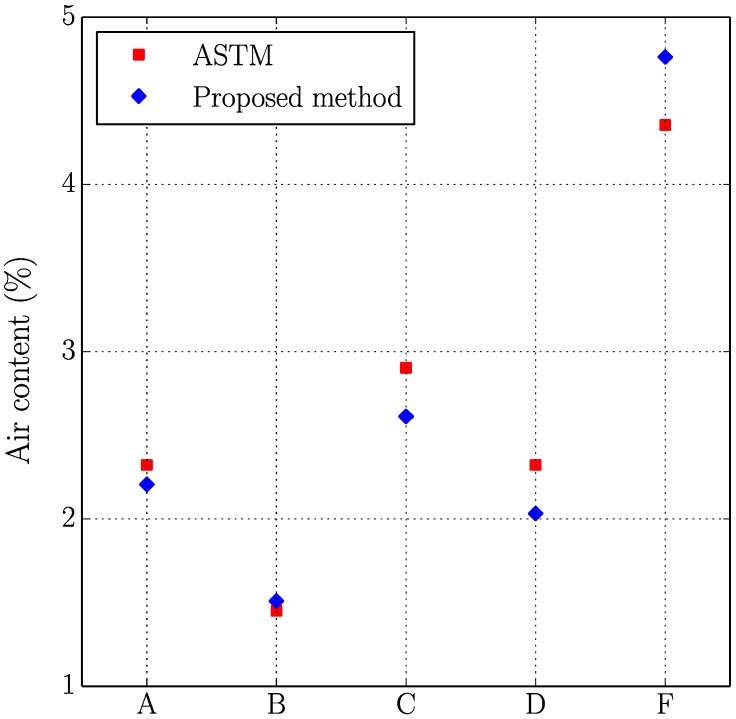
Results of the air content (%) of the slabs studied using the original and the proposed methods.

**Figure 10:**
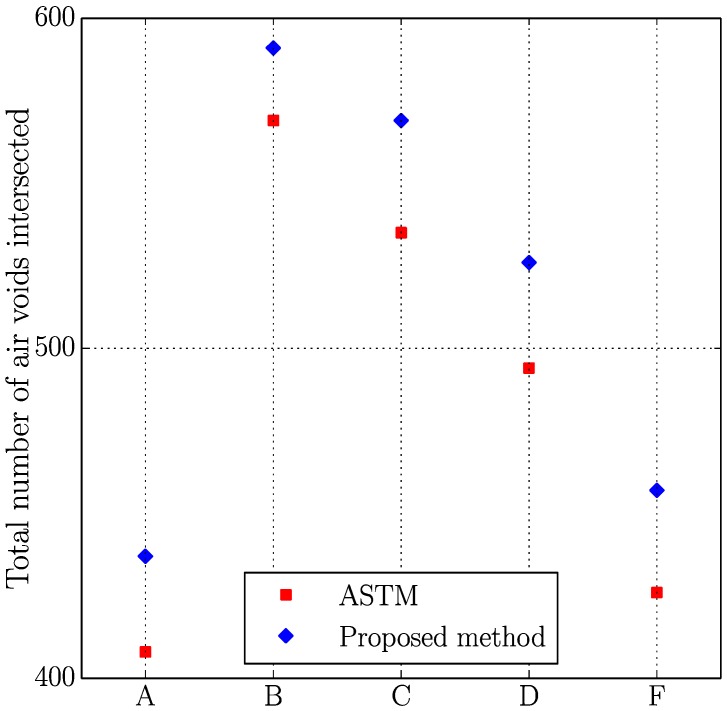
Results of the voids intersected of the slabs studied using the original and the proposed methods.

**Figure 11:**
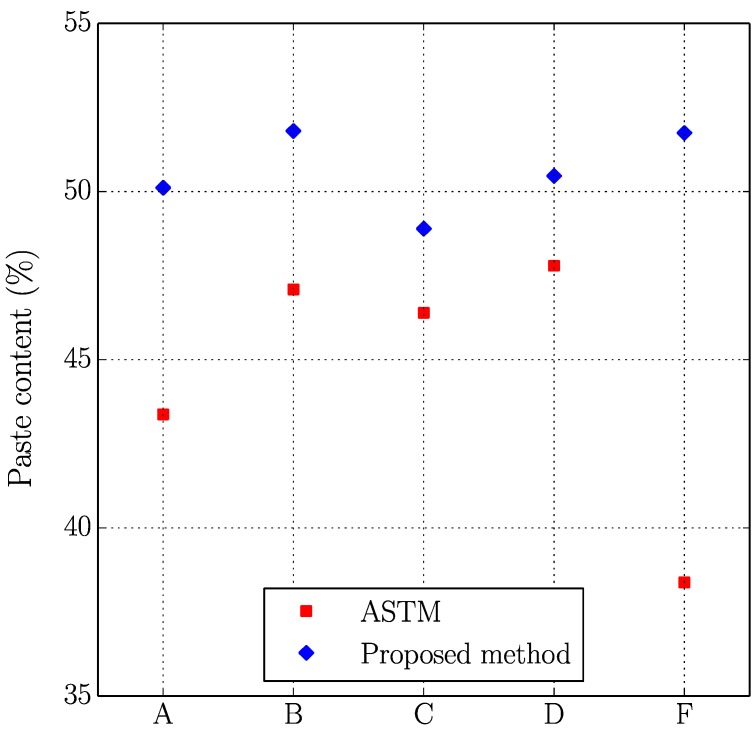
Results of the paste content (%) of the slabs studied using the original and the proposed methods.

**Figure 12:**
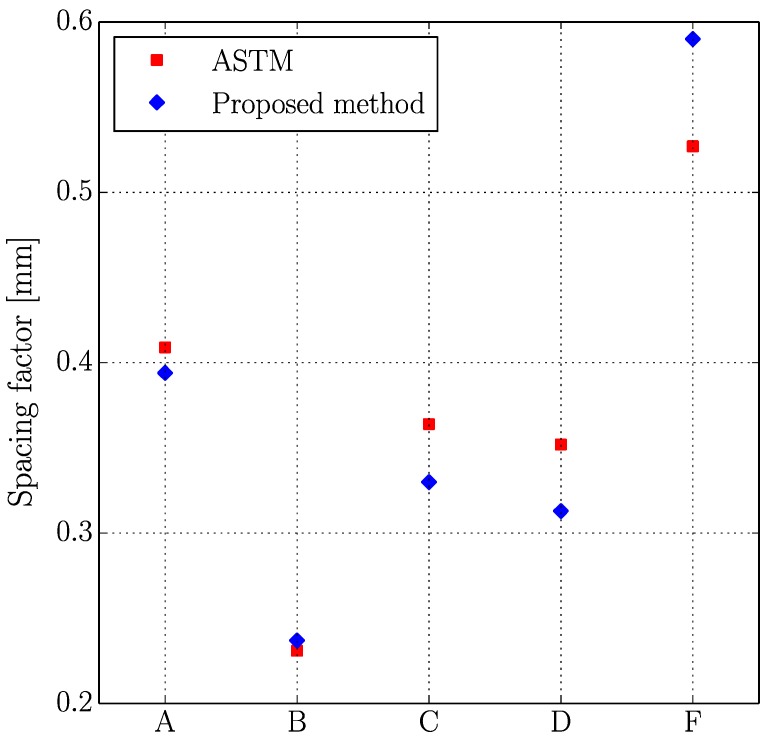
Results of the spacing factor of the slabs studied using the original and the proposed methods.

Table 6 shows the results of the air content (%), voids intersected/cm, paste content (%), and spacing factor obtained with both methodologies, which are plotted in Figure 9, Figure 10, Figure 11 and Figure 12. 

Similar comments as those made in the previous study (two operators using the proposed method) can be made here. Again, the results seem to correlate reasonably well between both methodologies, especially if air content (%), voids intersected/cm and spacing factor results are observed. It is true that differences are more important in the case of paste content (%), which is probably due to the fact that in some occasions grey aggregates can be confused with paste. Thus, the proposed method seems to agree well with the traditional method for different concrete mixtures with different air content and voids distribution. It is interesting to keep in mind that for each sample the same region of interest has been used, but this does not mean that exactly the same points of the virtual grid are used for determining the number of intersections with voids, paste or aggregates. This explains why, for example, the proposed method gives a higher air content than the traditional method for samples B and F but lower for the rest.

## 4. Conclusions

Macro photography as an alternative to stereoscopic microscope observation for determining the parameters that characterize the air-void system defined in the ASTM C457 Standard [8] has been successfully applied to a large set of hardened concrete specimens.

The methodology that is proposed here presents the following advantages when compared with the use of a stereoscopic microscope as described by the standard:

- A more comfortable procedure for the operator. The tedious task of identifying voids, aggregates and cement paste in the surface of analysis can be performed by using a computer screen instead of a stereoscopic microscope.
- A significant reduction in equipment cost.
- The convenience of saving the photographic compositions of the marked surface should it be necessary to repeat the observation. This avoids the need of saving and maintaining the concrete slabs in the proper conditions.

The composed pictures of the surface areas of each slab studied were checked so that they reached a high-enough resolution for the observation. In addition, the dispersion of the results of two slabs of the same dosage and those that were observed by two operators were within the range acceptable in comparison with the study described in the ASTM C457 Standard.

The proposed methodology has been compared with the original methodology described in the ASTM C457 Standard and has provided similar results, therefore, proving to be a reliable methodology for analysing the air-void system in concrete specimens.

As a final point, it should be noted that, while the procedure described here corresponds to the so-called Procedure B: the modified point-count method of the standard, it could also be used to apply the Procedure A: linear traverse method, since once the final image of the surface is obtained, the corresponding measurements can also be obtained.

## Figures and Tables

**Table 1:** Average precision of the assessment made by one operators on a set of 54 specimens (27 dosages with two slabs per dosage). The complete results can be found in the Appendix A at the end of the study.

Item	Standard Deviation (1s)	Range of Two Test Results (d2s)	Percent of Average	
			Coefficient of Variation (1s%)	Range of Two Test Results (d2s%)
Air content %:	0.80	2.27	13.8	39.1
Voids intersected/cm [in.]:	0.16	0.46	6.6	18.5
Paste content, %	1.65	4.65	3.9	10.9
Spacing factor	0.02	0.07	7.5	21.2

**Table 2:** Comparison of values obtained on 10 specimens (five dosages with two slabs per dosage) by two operators using the method proposed in this study.

Item	Air Content (%)		Air Voids Intersected		Paste Content (%)		Spacing Factor	
	Op. 1	Op. 2	Op. 1	Op. 2	Op. 1	Op. 2	Op. 1	Op. 2
1A	4.762	4.065	616	549	35.075	34.611	0.368	0.376
1B	5.110	4.530	564	537	32.346	34.495	0.402	0.407
2A	6.039	5.459	931	852	34.843	37.631	0.276	0.296
2B	5.226	4.123	731	628	34.669	36.585	0.324	0.340
3A	2.149	3.020	896	931	50.581	49.245	0.190	0.220
3B	3.717	4.472	848	858	50.348	49.535	0.274	0.298
4A	4.588	5.633	393	398	49.884	46.922	0.662	0.712
4B	4.762	5.517	335	342	48.432	46.051	0.782	0.812
5A	3.717	4.762	326	360	43.612	43.206	0.668	0.691
5B	2.904	3.078	340	361	44.541	44.077	0.580	0.546

**Table 3:** Average precision of the assessment made by two operators on a set composed by ten specimens.

Item	Standard Deviation (1s)	Range of Two Test Results (d2s)	Percent of Average	
			Coefficient of Variation (1s%)	Range of Two Test Results (d2s%)
Air content %:	0.52	1.48	10.9	30.8
Voids intersected/cm [in.]:	0.14	0.40	7.1	20.2
Paste content, %	1.29	3.66	3.7	10.4
Spacing factor	0.01	0.02	2.6	7.5

**Table 4:** Comparison of the average precision data of the air content (%) between this study and the European study submitted to ASTM C457 [8].

Research	Standard Deviation (1s)	Range of Two Test Results (d2s)
Results in this study	0.54	1.48
Results of the slabs prepared in originating lab and measured in originating lab submitted to ASTM C457	0.57	1.61
Results of the slabs prepared in originating lab but measured in participating labs from the European study submitted to ASTM C457	0.71	2.01

**Table 5:** Average precision data of the voids spacing obtained in this study compared with the European study submitted to ASTM C457 [8].

Research	Percent of Average	
	Coefficient of Variation (1s%)	Range of Two Test Results (d2s%)
Results in this study	7.5	21.2
Results of the slabs prepared in originating lab and measured inoriginating lab submitted to ASTM C457	8.0	22.6
Results of the slabs prepared in originating lab but measured inparticipating labs from the European study submitted to ASTM C457	20.1	56.9
Results of the slabs lapped and measured in participating labsfrom the European study submitted to ASTM C457	17.5	49.5

**Table 6:** Comparison of values obtained on five specimens by using a stereoscopic microscope (ASTM C457) and the method proposed in this study.

Specimen	Air Content (%)		Air Voids Intersected		Paste Content (%)		Spacing Factor	
	ASTM C457	Proposed Method	ASTM C457	Proposed Method	ASTM C457	Proposed Method	ASTM C457	Proposed Method
A	2.323	2.207	408	437	43.380	50.116	0.409	0.394
B	1.452	1.510	569	591	47.096	51.800	0.231	0.237
C	2.904	2.613	535	569	46.400	48.897	0.364	0.330
D	2.323	2.033	494	526	47.793	50.465	0.352	0.313
E	4.355	4.762	426	457	38.386	51.742	0.527	0.590

## Appendix A

Table A1, Table A2, Table A3 and Table A4 show the results for each of the 27 dosages used in the analysis of the proposed methodology by one operator (Section 3.2.1).

**Table A1:** Average value and dispersion data of the spacing factor of the 27 dosages (two slabs per dosage).

Dosage Denomination	Average Value of Spacing factor	Standard Deviation (1s)	Range of Two Test Results (d2s)	Coefficient of Variation (1s%)	Range of Two Test Results (d2s%)
C1	0.385	0.02	0.07	6.2	17.7
C2	0.300	0.03	0.10	11.3	32.0
C3	0.384	0.00	0.00	0.3	0.8
C4	0.401	0.07	0.20	17.5	49.6
C5	0.349	0.04	0.10	10.1	28.4
C6	0.472	0.03	0.10	7.4	20.9
C7	0.346	0.04	0.13	12.9	36.6
C8	0.352	0.02	0.05	4.9	13.8
C9	0.421	0.03	0.07	6.1	17.3
C10	0.314	0.03	0.10	11.0	31.2
C11	0.313	0.04	0.10	11.7	33.1
C12	0.245	0.00	0.01	1.5	4.2
C13	0.290	0.02	0.07	8.4	23.6
C14	0.314	0.01	0.02	2.7	7.6
C15	0.324	0.03	0.09	9.6	27.2
C16	0.321	0.03	0.07	8.2	23.1
C17	0.288	0.00	0.01	1.2	3.5
C18	0.280	0.01	0.03	3.3	9.3
C19	0.299	0.02	0.06	7.1	20.1
C20	0.282	0.04	0.10	13.0	36.9
C21	0.319	0.02	0.05	5.6	15.7
C22	0.287	0.02	0.04	5.4	15.3
C23	0.265	0.01	0.04	5.1	14.4
C24	0.292	0.05	0.13	15.5	43.8
C25	0.277	0.01	0.04	5.4	15.2
C26	0.309	0.01	0.03	3.0	8.4
C27	0.301	0.02	0.07	8.2	23.3
Average		0.02	0.07	7.5	21.2

**Table A2:** Average value and dispersion data of the voids intersected /cm of the 27 dosages (two slabs per dosage).

Dosage Denomination	Average Value of Voids Intersected/cm	Standard Deviation (1s)	Range of Two Test Results (d2s)	Coefficient of Variation (1s%)	Range of Two Test Results (d2s%)
C1	1.71	0.107	0.30	6.2	17.6
C2	2.41	0.411	1.16	17.0	48.1
C3	2.37	0.115	0.33	4.8	13.7
C4	1.61	0.082	0.23	5.1	14.5
C5	2.28	0.294	0.83	12.9	36.4
C6	1.70	0.004	0.01	0.2	0.7
C7	2.38	0.111	0.31	4.7	13.2
C8	2.39	0.055	0.16	2.3	6.6
C9	1.92	0.386	1.09	20.1	56.9
C10	2.71	0.023	0.06	0.8	2.4
C11	2.34	0.049	0.14	2.1	6.0
C12	2.23	0.014	0.04	0.6	1.8
C13	2.75	0.072	0.20	2.6	7.4
C14	2.69	0.119	0.34	4.4	12.5
C15	2.52	0.096	0.27	3.8	10.8
C16	2.64	0.037	0.10	1.4	4.0
C17	2.46	0.062	0.17	2.5	7.1
C18	2.94	0.388	1.10	13.2	37.3
C19	2.96	0.368	1.04	12.4	35.1
C20	3.05	0.244	0.69	8.0	22.7
C21	2.77	0.302	0.85	10.9	30.8
C22	2.66	0.125	0.35	4.7	13.3
C23	2.68	0.039	0.11	1.5	4.1
C24	2.92	0.129	0.37	4.4	12.5
C25	3.22	0.066	0.19	2.0	5.8
C26	2.30	0.435	1.23	18.9	53.5
C27	2.59	0.236	0.67	9.1	25.8
Average		0.162	0.46	6.6	18.5

**Table A3:** Average value and dispersion data of the air content (%) of the 27 dosages (two slabs per dosage).

Dosage Denomination	Average Value of Air Content	Standard Deviation (1s)	Range of Two Test Results (d2s)	Coefficient of Variation (1s%)	Range of Two Test Results (d2s%)
C1	4.94	0.246	0.70	5.0	14.1
C2	5.63	0.575	1.63	10.2	28.9
C3	6.85	0.739	2.09	10.8	30.5
C4	3.83	0.575	1.63	15.0	42.4
C5	5.40	0.329	0.93	6.1	17.2
C6	5.23	0.493	1.39	9.4	26.7
C7	6.62	1.314	3.72	19.8	56.1
C8	5.66	0.205	0.58	3.6	10.3
C9	5.37	1.519	4.30	28.3	80.0
C10	6.07	1.191	3.37	19.6	55.5
C11	4.67	1.437	4.07	30.7	87.0
C12	2.76	0.123	0.35	4.5	12.6
C13	5.14	0.534	1.51	10.4	29.4
C14	6.45	0.082	0.23	1.3	3.6
C15	5.81	0.411	1.16	7.1	20.0
C16	6.36	0.452	1.28	7.1	20.1
C17	4.91	0.041	0.12	0.8	2.4
C18	6.39	1.560	4.41	24.4	69.1
C19	7.06	0.945	2.67	13.4	37.9
C20	6.88	0.616	1.74	8.9	25.3
C21	7.08	1.643	4.65	23.2	65.6
C22	5.78	0.945	2.67	16.3	46.2
C23	5.40	0.575	1.63	10.6	30.1
C24	6.79	2.217	6.27	32.6	92.3
C25	7.38	0.411	1.16	5.6	15.8
C26	4.70	1.068	3.02	22.7	64.2
C27	5.60	1.437	4.06	25.6	72.5
Average		0.803	2.27	13.8	39.1

**Table A4:** Average value and dispersion data of the paste content (%) of the 27 dosages (two slabs per dosage).

Dosage Denomination	Average Value of Paste Content	Standard Deviation (1s)	Range of Two Test Results (d2s)	Coefficient of Variation (1s%)	Range of Two Test Results (d2s%)
C1	33.71	1.930	5.46	5.7	16.2
C2	34.76	0.123	0.35	0.4	1.0
C3	46.37	0.780	2.21	1.7	4.8
C4	43.15	4.024	11.38	9.3	26.4
C5	45.50	0.534	1.51	1.2	3.3
C6	48.40	2.094	5.92	4.3	12.2
C7	38.88	2.176	6.16	5.6	15.8
C8	48.64	0.616	1.74	1.3	3.6
C9	47.85	1.807	5.11	3.8	10.7
C10	46.49	1.109	3.14	2.4	6.7
C11	46.66	3.655	10.34	7.8	22.2
C12	45.62	1.766	4.99	3.9	10.9
C13	49.13	0.000	0.00	0.0	0.0
C14	42.31	0.862	2.44	2.0	5.8
C15	44.37	1.889	5.34	4.3	12.0
C16	43.15	2.874	8.13	6.7	18.8
C17	39.66	0.657	1.86	1.7	4.7
C18	40.65	2.874	8.13	7.1	20.0
C19	41.84	1.848	5.23	4.4	12.5
C20	40.22	0.041	0.12	0.1	0.3
C21	41.81	3.367	9.52	8.1	22.8
C22	38.94	0.945	2.67	2.4	6.9
C23	35.63	0.452	1.28	1.3	3.6
C24	41.20	1.519	4.30	3.7	10.4
C25	40.45	0.287	0.81	0.7	2.0
C26	42.07	3.326	9.41	7.9	22.4
C27	42.22	2.874	8.13	6.8	19.3
Average		1.646	4.65	3.9	10.9
